# Complement activation and increased anaphylatoxin receptor expression are associated with cortical grey matter lesions and the compartmentalised inflammatory response of multiple sclerosis

**DOI:** 10.3389/fncel.2023.1094106

**Published:** 2023-03-22

**Authors:** Rhian Evans, Lewis M. Watkins, Kristen Hawkins, Gabriella Santiago, Constantinos Demetriou, Michelle Naughton, Marie Dittmer, Mark I. Rees, Denise Fitzgerald, B. Paul Morgan, James W. Neal, Owain W. Howell

**Affiliations:** ^1^Faculty of Medicine, Health and Life Sciences, Swansea University Medical School, Swansea, United Kingdom; ^2^The Wellcome-Wolfson Institute for Experimental Medicine, Queen’s University Belfast, Belfast, United Kingdom; ^3^Centre for Experimental Medicine, Queen’s University Belfast, Belfast, United Kingdom; ^4^Faculty of Medicine and Health, The University of Sydney, Darlington, NSW, Australia; ^5^School of Medicine, UK Dementia Research Institute Cardiff and Systems Immunity Research Institute, Cardiff University, Cardiff, United Kingdom

**Keywords:** complement, demyelination, leptomeninges, microglia, inflammation

## Abstract

**Background:**

The extent of cortical pathology is an important determinant of multiple sclerosis (MS) severity. Cortical demyelination and neurodegeneration are related to inflammation of the overlying leptomeninges, a more inflammatory CSF milieu and with parenchymal microglia and astroglia activation. These are all components of the compartmentalised inflammatory response. Compartmentalised inflammation is a feature of progressive MS, which is not targeted by disease modifying therapies. Complement is differentially expressed in the MS CSF and complement, and complement receptors, are associated with demyelination and neurodegeneration.

**Methods:**

To better understand if complement activation in the leptomeninges is associated with underlying cortical demyelination, inflammation, and microglial activation, we performed a neuropathological study of progressive MS (*n* = 22, 14 females), neuroinflammatory (*n* = 8), and non-neurological disease controls (*n* = 10). We then quantified the relative extent of demyelination, connective tissue inflammation, complement, and complement receptor positive microglia/macrophages.

**Results:**

Complement was elevated at the leptomeninges, subpial, and within and around vessels of the cortical grey matter. The extent of complement C1q immunoreactivity correlated with connective tissue infiltrates, whilst activation products C4d, Bb, and C3b associated with grey matter demyelination, and C3a receptor 1+ and C5a receptor 1+ microglia/macrophages closely apposed C3b labelled cells. The density of C3a receptor 1+ and C5a receptor 1+ cells was increased at the expanding edge of subpial and leukocortical lesions. C5a receptor 1+ cells expressed TNFα, iNOS and contained puncta immunoreactive for proteolipid protein, neurofilament and synaptophysin, suggesting their involvement in grey matter lesion expansion.

**Interpretation:**

The presence of products of complement activation at the brain surfaces, their association with the extent of underlying pathology and increased complement anaphylatoxin receptor positive microglia/macrophages at expanding cortical grey matter lesions, could represent a target to modify compartmentalised inflammation and cortical demyelination.

## Introduction

Cortical demyelination and neurodegeneration are associated with a more severe and disabling multiple sclerosis (MS) (Calabrese et al., 2015; Absinta et al., 2020). Infiltrates of immune cells in the leptomeningeal and perivascular spaces, which are a feature of the broader compartmentalised inflammatory response that characterises established MS (Lassmann, 2019), associate with the extent of cortical pathology (Magliozzi et al., 2007; Reynolds et al., 2011; Lassmann, 2019; Kee et al., 2022). These cellular infiltrates represent an intrathecal source of soluble cytokines and inflammatory signals, which may activate microglia and astrocytes, so they become damaging to myelin or directly cytotoxic to neurons in the subpial and perivascular tissues (Gardner et al., 2013; Magliozzi et al., 2019b). Recognising the biological pathways associated with cortical pathology and compartmentalised inflammation will be important to guide future attempts to target such clinically meaningful aspects of MS pathology.

Inflammation of the leptomeninges and perivascular space is associated with gradients of tissue injury, detectable on advanced MRI and at autopsy, that represent substantial neuronal and glial alterations, and are most pronounced in the subpial tissue but extend through the depth of the cortical grey matter (GM) (Peterson et al., 2001; Magliozzi et al., 2010; Mainero et al., 2015; Griffiths et al., 2020; Junker et al., 2020). A transcriptomic and biochemical signature of inflammation, enriched for cytokines that support tissue-homing and lymphoid neogenesis, alongside complement proteins, characterise the cerebrospinal fluid (CSF) of cases with more extensive cortical GM lesions at diagnosis. A finding replicated in cases characterised by extensive cortical demyelination and leptomeningeal inflammation at post-mortem (Magliozzi et al., 2018, 2019a,b, 2020). Complement proteins (including C1q, C3), activation products (C4d) and their receptors, including anaphylatoxin receptors (C3aR1 and C5aR1), are located at subpial, leukocortical, white matter (WM) and deep GM areas in MS, where they associate with the extent of myelin and neuronal loss (Breij et al., 2008; Barnett et al., 2009; Ingram et al., 2014; Michailidou et al., 2015, 2017; Watkins et al., 2016; Loveless et al., 2018; Cooze et al., 2022). The concentration of complement proteins in the CSF differs between MS and non-inflammatory controls and between MS subtypes, disease severity and outcome (Aeinehband et al., 2015; Håkansson et al., 2020). Complement and complement receptors are essential to B: T-cell co-stimulation, monocyte: lymphocyte interactions and lymphocyte chemotaxis (West et al., 2018). Complement activation at the blood-CSF and CSF-brain barriers of the choroid epithelium, ependyma, pia mater and vascular endothelia are a feature of neuromyelitis optica, and complement activation is noted at the choroid plexus and in acute WM lesions in MS (Compston et al., 1989; Wayne Moore et al., 2016; Guo et al., 2017). It is not clear whether raised complement proteins in the CSF are accompanied by complement activation at CSF—blood brain barriers of the leptomeninges, pia and vasculature, or if complement activation at these brain barriers is associated with inflammatory changes in the underlying cortical GM.

Complement activation results in generation of anaphylatoxins C3a and C5a, which bind to G-protein-coupled anaphylatoxin receptors C3aR1 and C5aR1 (CD88) expressed by monocytes, macrophages, microglia, and some activated astrocytes (Müller-Ladner et al., 1996; Gasque et al., 1997, 1998; Ischenko et al., 1998). Complement anaphylatoxins are raised in MS CSF (Ingram et al., 2010; Håkansson et al., 2020) and CSF C5a levels and C5a inhibition in the CSF ameliorates blood-brain barrier disruption (Faustmann et al., 1995; Flierl et al., 2009). Activation of C3aR1 and C5aR1 on immune cells promote upregulation of functional pathways relevant to inflammation (including oxidative burst), the release of soluble chemoattractant and pro-inflammatory cytokines [including tumour necrosis factor α (TNFα) and interferon γ] and stimulates phagocytosis and metabolic dysfunction (Haas and van Strijp, 2007; Klos et al., 2009; Vandendriessche et al., 2021). The extent of experimentally induced demyelination and inflammation is enhanced in mice overexpressing C3a and C5a, and deletion of C5aR1 or the application of C5aR1 antagonists, reduces the severity of injury (Nataf et al., 1998, 1999; Ingersoll et al., 2010; Peterson et al., 2017). These findings indicate the potential value of targeting the C5a—C5aR1 axis to improve disease outcome.

To better understand the contribution of complement activation and anaphylatoxin receptor expressing cells to processes of compartmentalised inflammation and cortical pathology, our post-mortem study of MS investigated: (i) the presence of products of complement activation at the brain surfaces (pia and around blood vessels of the cortex); (ii) the association between complement, microglial and astroglial activation and; (iii) differences in complement C3aR1 and C5aR1 positive cell numbers in areas of cortical demyelination in comparison to non-lesion and control GM samples. We found that complement at the brain surfaces associated with the extent of compartmentalised inflammation, cortical demyelination and complement anaphylatoxin receptor positive microglia at the expanding lesion edge. Collectively, our findings add to the weight of evidence implicating a key role for complement activation at the brain surface and complement-microglia interactions *via* C5aR1 to the pathological severity of MS.

## Materials and methods

### Post-mortem tissue

We used cryopreserved cortical tissue sections containing cortical GM and subcortical WM from regions of frontal, temporal and parietal lobes of the forebrain from post-mortem cases of progressive MS [*n* = 22; provided by the UK MS Tissue Bank (MSTB), Imperial College London], non-neurological disease controls (NNDC; *n* = 13), and inflammatory disease controls (*n* = 8; provided by the UK MSTB and Oxford tissue bank) with appropriate ethical approval (08/MRE09/31 + 5 and 13/WA/0292). Please see Table 1 for further details and demographics.

**TABLE 1:** Summary of MS, non-neurological disease controls, and inflammatory disease controls (Inflam) used in this study.

Case MS	Sex	Age of death	Cause of death	PMD (hours)	MS type	MS disease Duration (years)	MS inflam. (1–3)
MS402	M	46	MS	12	SPMS	20	+++
MS405	M	62	MS	12	SPMS	25	+
MS407	F	44	MS	22	SPMS	19	+++
MS408	M	39	MS	21	SPMS	10	++
MS422	M	58	MS	25	SPMS	13	++
MS423	F	54	MS	11	SPMS	30	++
MS425	F	46	MS	25	SPMS	21	+
MS438	F	53	MS	17	SPMS	18	+++
MS444	M	49	MS	18	SPMS	20	++
MS473	F	39	MS	9	PPMS	13	++
MS485	F	57	MS	24	PPMS	29	+
MS491	F	64	MS	9	SPMS	25	+
MS492	F	66	MS	15	PPMS	31	+
MS497	F	60	MS	26	SPMS	29	+++
MS510	F	38	MS	19	SPMS	22	+++
MS513	M	51	MS	17	SPMS	18	++
MS517	F	48	MS	12	PPMS	25	+
MS523	F	63	MS	20	SPMS	32	+
MS527	M	47	MS	10	SPMS	25	0
MS528	F	75	MS	17	SPMS	25	++
MS530	M	42	MS	15	SPMS	24	+++
MS538	F	61	MS	12	SPMS	39	++
***N* = 22** **MS**	**8 M** **14 F**	**53** years (38–75)		17 h (9–26)	4 PPMS 18 SPMS	23 years (10–39)	
**Case** **Controls**	**Sex**	**Age of death**	**Cause of death**	**PMD (hours)**			
CO25	M	35	Carcinoma of the Tongue	22			
12/023	M	69	Unknown	24			
12/046	M	72	Unknown	24			
12/048	F	65	Ovarian cancer	48			
12/052	F	42	Pancreatic cancer	48			
12/088	M	51	Cardiac arrest	24			
11/093	F	52	Chronic liver disease	48			
11/122	F	65	Unknown	24			
12/132	F	67	Unknown	48			
1231/93	M	58	Unknown	n/a			
***N* = 10** **controls**	**5 M** **5 F**	58 years (35–72)		34 h (22–48)			
Case Inflam	Sex	Age of death	Cause of death	PMD (h)			
B4938	M	18	HSV encephalopathy	n/a			
C2342	M	17	HIV encephalopathy	24			
C3727	M	41	HIV encephalopathy	n/a			
C4178	M	59	CMV encephalopathy	n/a			
91/1343	M	32	Bronchopneumonia	48			
1140/95	F	65	Ischaemic encephalopathy	n/a			
1078/95	M	32	Ischaemic encephalopathy	24			
1062/00	F	49	Ischaemic encephalopathy	72			
***N* = 8** **Inflam controls**	**6 M** **2 F**	**40 years** (17–65)	**4 Viral encephalitis** **4 Ischaemic stroke**	42 h (24–72)			

Sex, age at death, cause of death and post-mortem delay (PMD) are reported where available. In addition, for the MS cohort, disease subtype at death (either SP or PPMS), duration of disease from first symptom onset, and the relative extent of leptomeningeal and perivascular inflammation (MS inflam; graded 0–3, as described in the methods) are reported. Text in bold summarises the number of cases and column means (and range) for each cohort. CMV, cytomegalovirus; F, female; HIV, Human immunodeficiency virus; HSV, herpes simplex virus; M, male; PMD, post-mortem delay; PPMS, primary progressive MS; SPMS, secondary progressive MS.

### Immunohistochemistry

Cryosections (8 μm thick) were incubated in hydrogen peroxide solution (Sigma-Aldrich), blocked with normal horse serum and incubated with the primary antibody overnight (Table 2 contains details of primary antibodies used). Sections were then incubated with a biotinylated species-specific secondary antibody prior to the addition of a peroxidase-linked avidin-biotin complex (ABC Elite, Vector Laboratories Ltd.) and immunostaining visualised with diamino-benzidine (DAB; Immpact DAB, Vector Laboratories Ltd.) as the chromogen. If a second antigen was to be detected in a dual immunostaining experiment (e.g., C5aR1 and proteolipid protein; PLP), then sections were briefly heated in Tris-EDTA buffer (20 min in a bench top steamer following the completed detection and DAB chromogen development) to remove the first immune complexes, prior to the addition of the second primary antibody of interest in preparation for the biotinylated-secondary antibody and tertiary amplification using an alkaline-phosphatase linked reporter enzyme and Vector Blue as the chromogen (Bauer and Lassmann, 2014). Cells were counterstained with haematoxylin or cresyl violet. Dependent on the chromogen(s) used, sections were either rinsed in H_2_O, dried, and mounted with VecaMount permanent mounting media (Vector labs.) or dehydrated through alcohol, cleared in xylene and depex mounted (Thermofisher Scientific). All sections from all cases were immunostained for a single or dual target as part of the same experiment and included primary antibody-negative controls and irrelevant species-specific antisera as positive controls. Images were taken with a Zeiss Axio Scope 1 at 100–630× magnification fitted with a Zeiss MRm 503 colour camera or with a Zeiss Axio Scanner 1 and handled in QuPath (Bankhead et al., 2017)^1^ or FUJI.^2^

**TABLE 2:** Primary antibodies used in this study.

Antigen	Host	Clone	Source
C1q	Rabbit	Polyclonal	Dako/agilent
C3b-iC3b	Mouse	C330	In-house (Cardiff University)
Fragment Bb	Mouse	Monoclonal IgG2	Pathway diagnostics
C4d	Mouse	Monoclonal IgG1	Pathway diagnostics
Myelin oligodendrocyte glycoprotein	Mouse	Y10	Imperial (Prof. Reynolds)
Proteolipid protein	Mouse	PLPC1	AbD Serotec/Merck
Human leukocyte antigens DP-DQ-DR	Mouse	Cr3/43	Dako/agilent
TMEM119	Rabbit	Polyclonal	Sigma
CD68	Mouse	KP1	Dako/agilent
Ionised calcium binding adapter molecule 1	Rabbit	Polyclonal	WAKO/Fujifilm
C5aR1/CD88	Rabbit	Polyclonal	BG Pharmingen
C3aR1	Mouse	hC3aRZ8	Hycult Biotech
Triggering receptor expressed on myeloid cells 2	Mouse	9D10	In-house (Cardiff University)
Microtubule associated protein 2	Mouse	Ap-20	Abcam
Neurofilament-H	Mouse	RT-97	Merck
Synaptophysin	Mouse	SY38	Merck
OLIG2	Rabbit	Polyclonal	Merck
Glial fibrillary acidic protein	Rabbit	Polyclonal	Dako/agilent
Tumour necrosis factor α	Goat	Polyclonal	R&D Systems
Inducible nitric oxide synthase	Mouse	2D2-B2	R&D Systems

Antibody name, target, clone, and source are listed.

### Neuropathological characterisation and quantitative analysis

For histological analysis, tissue was stained with luxol fast blue (LFB; ThermoFisher), and sequential sections immunohistochemically stained with anti- myelin oligodendrocyte protein (MOG) and anti- HLA-D. Cortical GM lesions were characterised based on their relative location (subpial, intracortical, leukocortical or cortex spanning—also termed type 4) and were further sub-categorised as chronic active or chronic inactive cortical GM lesions (no active lesions were noted) based on the presence of a visible rim of HLA-D and/or CD68+ microglia/macrophages (Peterson et al., 2001).

Areas of demyelination were measured from digitally scanned slides and lesions (GM or WM), total section and total GM and WM areas annotated and measured using QuPath. The percent area of demyelinated GM or WM was calculated relative to total grey or white matter in that sample and the mean percent lesion area per lesion type (subpial, cortex spanning—type 4 or leukocortical, WM lesion) calculated per case.

A manual quantification of GFAP and GFAP/C3b+ astrocytes was performed across the entire depth of the cortical GM, for each dual GFAP/C3b stained section. Six equal sized regions of interest (ROIs; total area 1.1 mm^2^) were placed perpendicularly across the neocortex starting at the pial surface and extending to the grey/WM border, to capture a report of the density of cells at superficial, medial, and more distal points of the cortex. All measurements were acquired at the margins of the sulci to ensure consistency of data collection. The density of astrocytes was investigated in cortical samples from cases with subpial lesions, normal appearing GM and control cortex.

A rating of relative leptomeningeal and perivascular cellular infiltration was generated for each case which reflected the most significant cellular infiltrate in the leptomeninges and/or perivascular space from a minimum of eight separate cortical tissue blocks per case. The extent of leptomeningeal inflammation was scored semi-quantitatively as: absent = 0; mild (+); moderate (++) and substantial (+++, including aggregates resembling follicle-like structures) in accordance with (Choi et al., 2012; Bevan et al., 2018; see Supplementary Figure 1).

Microglia/macrophage quantification (HLA-D, CD68, C3aR, C5aR1) was performed within GM lesions, NAGM and control GM in anatomically matched regions of subpial and deep cortical GM. Four 200× (area 0.239 mm^2^/field of view; Aperio, ImageScope) images were captured per lesion, anatomically matched NAGM and control GM. The number of immunopositive cells with an identifiable cell soma were quantified per ROI (average across the four fields of view) for comparison.

Morphological analysis of complement receptor expressing microglia/macrophages was performed on cells from the same GML ROI used for quantitative analysis (above). A 4 × 4 grid was overlaid onto the images and the entire cell body of complement receptor immunopositive cells was encapsulated using the wand tool in FIJI,^3^ whilst adjusting the threshold detection as necessary to ensure best fit. Five immunopositive cells were selected at random from each of the four images per ROI, per case (20 cells per ROI, 6 separate ROIs per MS case). Captured cell morphology was measured using the shape descriptors: Area; perimeter and circularity [4π × (Area)/(perimeter)^2^]. All values per cell were plotted and compared by Kruskal–Wallis and Dunn’s post-test.

### Semi-quantitative scoring of complement immunostaining

Cases of progressive MS, neuroinflammatory disease controls and non-neurological disease controls were immunostained for complement component C1q and complement activation products C3b/iC3b (herein termed C3b), C4d and Bb. The extent of complement immunoreactivity for each individual complement marker was rated between 0 and 5, as assessed from 400× images captured in quadruplicate across each ROI per cortical block for each case for MS, non-neurological disease control and neuroinflammatory-disease control cases, respectively. An assessment of complement at the leptomeninges and subpial was determined by the relative extent of positive staining of the connective tissue, meningeal blood vessels, pia mater and the relative number of immunopositive cells in the underlying subpial. The rating of complement immunostaining of the blood vessels in the GM and WM included an assessment of vessel and perivascular staining and the maximum number of complement + cells immediately adjacent to the blood vessels (please see Supplementary Table 1 for further details). Ratings were averaged per case for comparative analysis. Average case ratings were compared with the percent demyelination and connective tissue inflammation ratings for that case by non-parametric analysis as described in the results.

### *In situ* hybridisation

To detect transcripts of complement C3 (NM_000064.4) we used a 50 fluorescein (FAM)-labelled 19mer antisense oligonucleotide containing locked nucleic acid (LNA) and 2’-O-methyl RNA moieties at a 1:2 ratio using a previously described approach (Budde et al., 2008; Watkins et al., 2016). Probes; antisense C3 (FAM-TaaTccAccAauCauTucT) and sense C3 (FAM-AgaAauGauUggUggAuuA; where capitals indicate LNA and lower case 2’-O-Methyl RNA in all instances; Eurogentec, Southampton, UK). Hybridisation and wash conditions were optimised so that all sense probes yielded essentially no signal; sense probe, and no probe controls, were included in each experiment. Probe detection was performed on 10 μm thick frozen sections prepared from two controls (13/011 and 13/073) and four MS cases (MS422, MS425, MS438, MS527). Hybridised probe was visualised with a peroxidase-conjugated goat anti-FAM (Vector Laboratories) and DAB prior to anti-TMEM119 immunostaining, with detection with an anti-rabbit biotinylated secondary and an ABC-alkaline phosphate tertiary complex with Vector Blue as the chromogen (Vector Labs.).

### Generation of human monocyte-derived microglia-like cells

Peripheral blood mononuclear cells (PBMCs) were extracted from whole blood from healthy donors. Blood was layered onto an equal volume of lymphoprep (Stemcell Technologies) in 50 ml sterile falcon tubes, centrifuged at room temperature for 20 min at 2000 × *g*, and the buffy coat (layer containing PBMCs) was collected and gently transferred to a universal tube. The PBMCs were washed twice by centrifugation with pre-warmed media (RPMI-1640 with GlutaMax; Fisher Scientific), the pellet resuspended in 80 μl of ice-cold MACS buffer [phosphate buffered saline (PBS) containing 2% foetal bovine serum (FBS; Fisher Scientific)], and 20 μl CD14 magnetic microbeads (Miltenyi Biotec) added per 1 × 10^7^ cells. Cells were sorted twice by positive selection using an autoMACS pro (Miltenyi Biotec), the resultant CD14+ monocytes resuspended in medium (RPMI-1640 with glutaMAX containing 10% FBS and 1% pen-strep; Fisher Scientific) and plated onto poly-L-lysine (100 μg/ml) coated 96-well plates, 100 μl/well, at a density of 1 × 10^5^ cells/well and left to adhere for 24 h. Differentiation of CD14+ monocytes to microglia-like cells was achieved by incubation in the above medium containing 0.1 μg/ml IL-34 and 0.01 μg/ml granulocyte-macrophage colony-stimulating factor (Bio-Techne). Every other day, half the medium was aspirated and replaced with fresh cytokine containing medium. Cells were paraformaldehyde (PFA)-fixed for immunocytochemistry on day 10.

### Generation of primary murine dissociated neural cultures

Dissociated cultures of primary murine CNS tissue were generated from whole brains of day seven postnatal C57BL/6 pups (Dittmer et al., 2018). All animal maintenance and experiments followed the UK home office and approved by the Universities ethics committee (Queen’s University Belfast). Pups were sacrificed by decapitation, brain harvested, olfactory bulbs removed from the dissected whole brain tissue before cortical, cerebellar, and brain-stem tissue was minced, dissociated, washed and resuspended in mixed glial media (Dulbecco’s modified essential media; 10% low endotoxin foetal calf serum; 1% pen-strep; 1% L-glutamine) at 6.67 × 10^5^ cells/ml and seeded in 150 μl media per well (1 × 10^5^ cells/well) onto poly-L-lysine coated (10 μg/ml; Sigma) black flat-bottom 96-well plates (Thermofisher). Once plated, cells were cultured at 37°C, 5% CO_2_ for 5 days and medium was replenished on days 1, 3, and 5. Cell cultures were directly fixed with 4% PFA solution for 15 min after medium removal for immunocytochemistry as described (Dittmer et al., 2018).

### Data handling and statistical analysis

Data was handled and visualised in GraphPad Prism (Version 9). D’Agostino-Pearson normality testing revealed the majority of the data to be non-parametric. Results were presented as box and whisker plots showing minimum to maximum values, interquartile range, and group medians. Two-group comparisons, for example, in comparing the extent of GM and WM demyelination, were made using the non-parametric Mann–Whitney *t*-test. Three or more groups, for example, when comparing the semi-quantitative complement ratings from immunohistochemical analysis of leptomeningeal and pial/subpial tissue, perivascular sites of cortical GM and subcortical WM, relative to control and inflammatory disease controls, used the Kruskal–Wallis and Dunn’s multiple adjusted post-tests. Associations between the extent of complement immunostaining and demyelination were investigated by Spearman’s analysis. Statistical significance was set at *p* < 0.05 (*), *p* < 0.01 (^**^), *p* < 0.001 (^***^), and *p* < 0.0001 (^*⁣*⁣**^) .

## Results

### Complement is activated in brain connective tissue spaces and parenchyma and associates with the extent of compartmentalised inflammation and cortical demyelination

We sought to investigate the relationship between complement activation, compartmentalised inflammation, and demyelination in progressive MS. Our cohort of 22 progressive MS cases was representative of the inflammatory and actively worsening pathology of chronic disease. Cases harboured widespread microglia/macrophage activation, connective tissue infiltrates and displayed a variable abundance of cortical GM and subcortical WM demyelinating lesions (Supplementary Figure 1 and Table 1). The median rating for connective tissue inflammation was ++ (range 0 to +++), with 6/22 (27.3%) cases rated +++ (substantial immune cell infiltration and/or tertiary lymphoid-like structures; Supplementary Figures 1D, E). The median percentage lesion area was 19.2% (range 0–88.3%) of total GM for GM lesions and 16.6% (0–77.4%) of total WM for WM lesions. There were 64 lesions in total, with 53 GM lesions and 11 WM lesions. Cortical GM lesions were predominantly characterised as chronic inactive (C/I), and 60.3% (32 of 53) GM lesions were subpial lesions (Supplementary Figures 1F, G).

**FIGURE 1:**
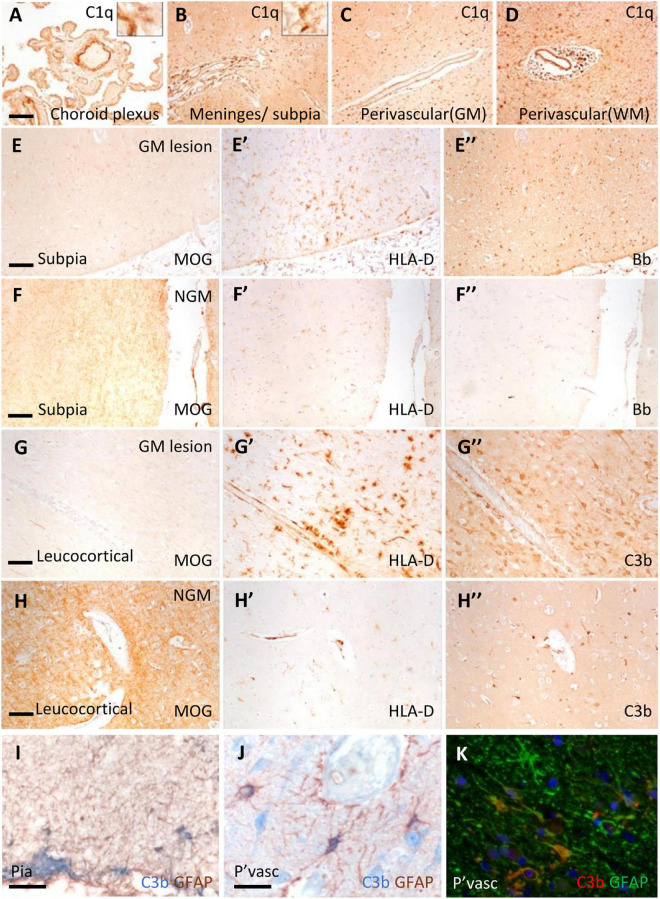
Complement recognition molecule C1q and activation products decorated brain barriers of the choroid plexus, leptomeninges, and perivascular spaces. Representative example of C1q immunostaining of the choroid plexus, leptomeninges, and perivascular cuffs and more diffusely in the tissue parenchyma of both the white (WM) and grey matter (GM; **A–D**). Complement Bb **(E–F”)**, and C3b **(G–H”)** immunoreactivity was elevated at sites of subpial and leukocortical GM lesions in comparison to normal appearing GM (NAGM; MOG and HLA-D immunostaining) from the same case **(E–E”)**. Dual-immunostaining revealed C3b + subpial astrocytes (GFAP+) and neuron-like cells at the glial-limitans at vascular and subpial sites **(I,J)**. C3b + /GFAP + astrocytes in the deeper laminae of the cortical GM **(K)**. Scale bars panels **(A–D)** = 100 μm; panels **(E–I)** = 50 μm; panels **(J,K)** = 25 μm.

Immunostaining revealed C1q and complement activation products in the choroid plexus, leptomeninges (including pia mater), and parenchyma of cortical GM and subcortical WM (Figure 1). Examples of complement recognition molecule C1q immunoreactivity decorating the choroid epithelium and vasculature, cells of the leptomeninges and parenchyma at subpial and perivascular sites of the underlying neocortex are shown (Figures 1A–D). Immunoreactivity for complement activation fragments Bb and C3b was variable, marking subpial and parenchymal sites near vessels of the GM (Figures 1E”–H”), in broad accordance with the pattern of demyelination (anti-MOG; Figures 1E–H) and the distribution and density of microglial/macrophages (anti- HLA-D; Figures 1E’–H’).

Microglial activation was associated with reactive morphological and phenotypic changes in astrocytes (Figures 1I–K). We observed GFAP/C3b+ astrocytes in close apposition with the glial limitans at subpial and perivascular sites of the progressive MS cortex (Figures 1I, J). C3b immunostaining was cytoplasmic, suggesting uptake of C3b-opsonised material, or the intracellular cleavage of C3 by astrocytes in the MS neocortex (Figure 1K). Quantification of GFAP and GFAP/C3b+ astrocytes in the normal appearing and demyelinated MS cortex revealed the loss of GFAP single positive astrocytes in areas of demyelination, whilst GFAP/C3b+ numbers were unchanged (Supplementary Figure 2) between control, non-lesioned, and lesioned GM.

**FIGURE 2:**
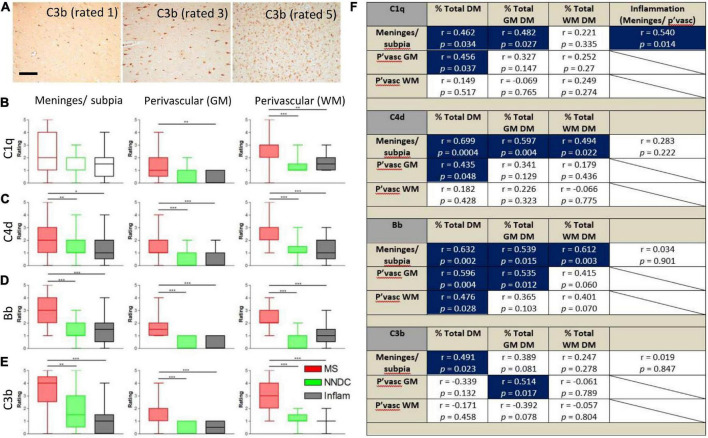
Semi-quantitative assessment of complement immunoreactivity in the progressive MS neocortex. Examples of complement C3b immunostaining rated as mild (1/5), modest (3/5), and substantial (5/5; **A**). Region-matched cortical tissue blocks from 22 progressive MS, 10 non-neurological controls (green bars) and 6 inflammatory disease controls (grey bars) were assessed for complement C1q, C4d, Bb, and C3b immunoreactivity at the meninges/subpial (left column) and perivascular sites of the grey (GM, centre column) and white mater (WM, right column of graphs; **B–E**). The relative abundance of C1q in the meninges/subpial correlated with the extent of underlying cortical demyelination and leptomeningeal inflammation; **F**), whilst expression of complement activation products C4d and Bb associated with the extent of GM demyelination (% total GM DM), and Bb abd C3b at and adjacent to vessels of the GM (P’vasc GM) correlated with the extent of GM demyelination (**F**; Blue areas indicate correlations with significant results). Group medians were compared with Kruskal–Wallis and Dunn’s multiple comparisons post-test and minimum and maximum values displayed. The extent of complement immunoreactivity was compared to GM pathology by Spearman correlation and *r*-values and *p*-values are shown for all comparisons. Pvasc, perivascular sites; % total DM, percent demyelination of the block, % total GM or WM, percent demyelination of the white or grey matter. Scale bar = 100 μm.

Semi-quantitative rating of complement recognition molecule C1q and products of complement activation C4d (classical and lectin/mannose binding pathways to activation), Bb (alternative pathway) and C3b (all pathways; Figure 2A) revealed increased complement immunoreactivity in progressive MS cases in comparison to non-neurological disease controls and to inflammatory disease controls (which were selected based on the presence of an anatomically matched tissue block that in most cases, did not harbour sites of extensive inflammatory pathology; Figures 2B–E and Supplementary Table 1). The relative extent of C1q immunoreactivity was increased at perivascular sites in cortical and subcortical areas of MS brain (Figure 2B), whilst the extent of C4d, Bb, and C3b immunoreactivity was increased in comparison to control and inflammatory disease controls at leptomeningeal/pial sites, and at perivascular/parenchymal sites in the GM and WM (Figures 2C–E).

By comparing assessments of complement immunoreactivity to measures of leptomeningeal cellular infiltrates, and the relative area of cortical and subcortical demyelination, we show that the extent of C1q immunoreactivity of the meninges/subpial was correlated with connective tissue inflammation; C4d and Bb immunoreactivity at the meninges/subpial was correlated with cortical demyelination (% total GM), whilst increased Bb and C3b at perivascular sites in the cortical GM correlated with the extent of GM demyelination (Spearman *r* ≥ 0.43, *p* < 0.05 in all instances; Figure 2F).

### C3aR1 and C5aR1 expression was widespread and associated with complement activation in cortical grey matter lesions

**FIGURE 3:**
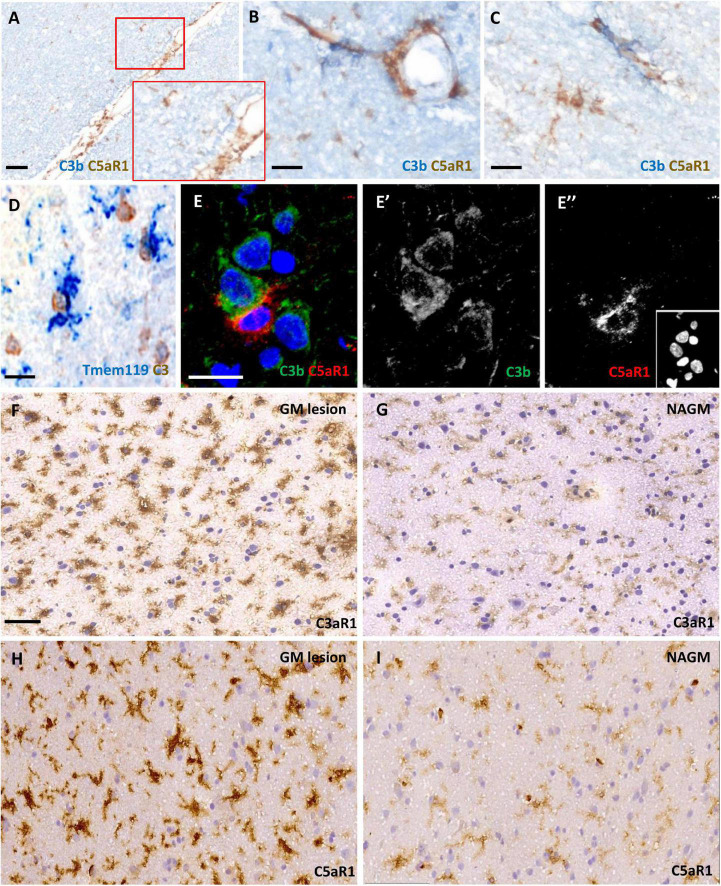
Anaphylatoxin receptor expression at sites of complement activation. Complement C3b (blue reaction product) and C5aR1 + cells (brown reaction product) and subpial and perivascular sites of the cortical GM **(A–C)**. TMEM119 + immunopositive microglia (blue) in close contact with large neuron-like cells expressing C3 transcript **(D)** and an example of a C5aR1 + cell contacting a C3b immunoreactive cortical neuron **(E–E”)**. Immunostaining revealed an elevated number and more darkly stained C3aR1 and C5aR1 + cells with a microglia-like morphology in the demyelinated cortical GM (GM lesion; **F,H**) in comparison to adjacent normal appearing GM (NAGM; **G,I**). Scale bars; panels **(A,F–I)** = 50 μm and panels **(B–E”)** = 20 μm.

We assessed C3aR1 and C5aR1 expression in samples of cortical GM (Figure 3). C5aR1+ amoeboid and ramified cells with a microglia/macrophage-like morphology of the cortical GM were associated with C3b immunoreactivity at the leptomeninges and perivascular spaces (Figures 3A–C). Resident (TMEM119+; blue reaction product) microglia were noted in close apposition to large cells expressing transcripts for C3 (brown reaction product), showing that some of this complement-specific signal is generated in the CNS (Figure 3D). Immunofluorescence revealed C5aR1+ cells near sites of C3b deposition (Figures 3E–E”). C3aR1 and C5aR1+ cells with a ramified, microglia-like morphology, were seen at variable densities across the demyelinated and normal- appearing cortical GM (Figures 3F–I).

### Microglia/macrophages express complement anaphylatoxin C3a and C5a receptors

**FIGURE 4:**
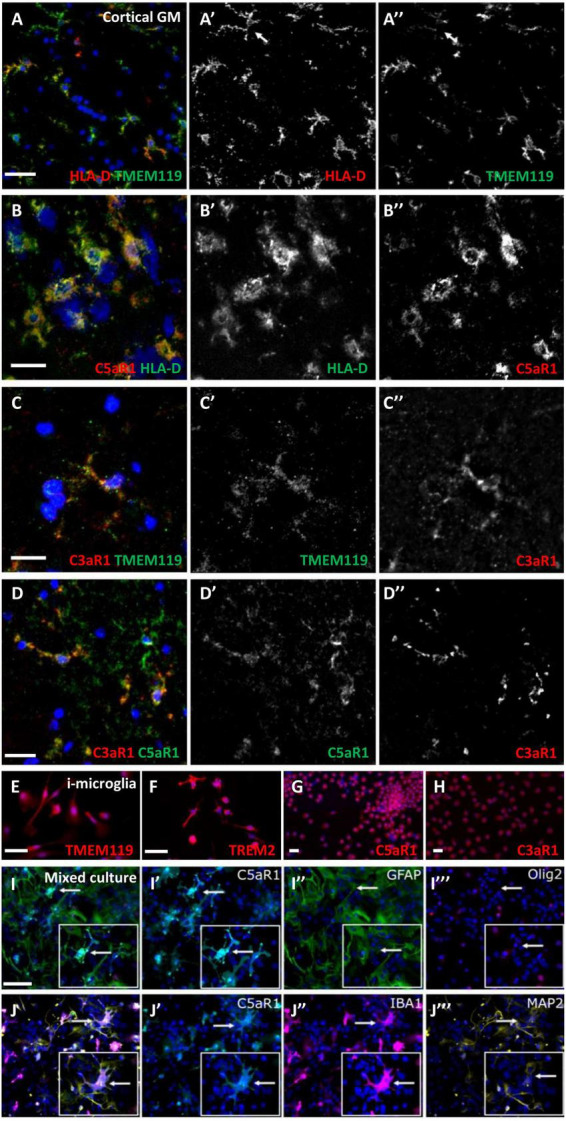
Microglia/macrophages express complement anaphylatoxin C3a and C5a receptors. C5aR1 microglia/macrophages were seen in the cortical GM in association with TMEM119 **(A–A”)** and HLA-D **(B–B”)**, and an example of C3aR1 + /TMEM119 + **(C–C”)**. Dual C3a/C5aR1 immunostaining showing co-positive C3aR1/C5aR1 cells **(D–D”)**. Human monocyte-derived microglia-like cells (i-microglia) were cultured for 10 days *in vitro* and expressed TMEM119 **(E)**, TREM2 **(F),** and C3aR1 and C5aR1 **(G,H)**. Multiplex immunocytochemistry of primary cultures from neonatal mouse brains revealed C5aR1 expression was associated with IBA-1 + microglia but not astrocytes, oligodendrocyte precursor cells (GFAP+, Olig2+, respectively, **I–J”**) or neurons (MAP2 + ; **J”’**, arrows represent cells shown in inserts) in day 5 cultures. Scale bars = 20 μm.

Frequently, cells with a microglia-like morphology, immunopositive for HLA-D and TMEM119, co-expressed C3aR1 or C5aR1 (Figures 4A–C”). Some microglia-like cells were C3aR1+/C5aR1+ (Figures 4D–D”). Staining of human monocyte-derived microglia-like cells that express microglia/macrophage markers, such as TMEM119 and TREM2 (Figures 4E, F), showed a robust and near-ubiquitous co-expression of C3aR1 and C5aR1 (Figures 4G, H). We generated primary dissociated cultures from the neonatal mouse brain and confirmed a microglia/macrophage expression of C5aR1 (co-localised with IBA-1+ cells; Figures 4J’, J”), whilst astrocytes (GFAP+), neurons (MAP2+), and oligodendrocyte-lineage cells (Olig2+) were negative for anti-C5aR1 immunoreactivity (Figures 4I–J”’) in day 5 cultures. As anticipated, these cultures represented a reliable source of expanding numbers of glial and neuronal cell populations (Supplementary Figure 3; Dittmer et al., 2018).

### The density of C5aR1 positive microglia/macrophages is increased at the expanding cortical grey matter lesion edge

**FIGURE 5:**
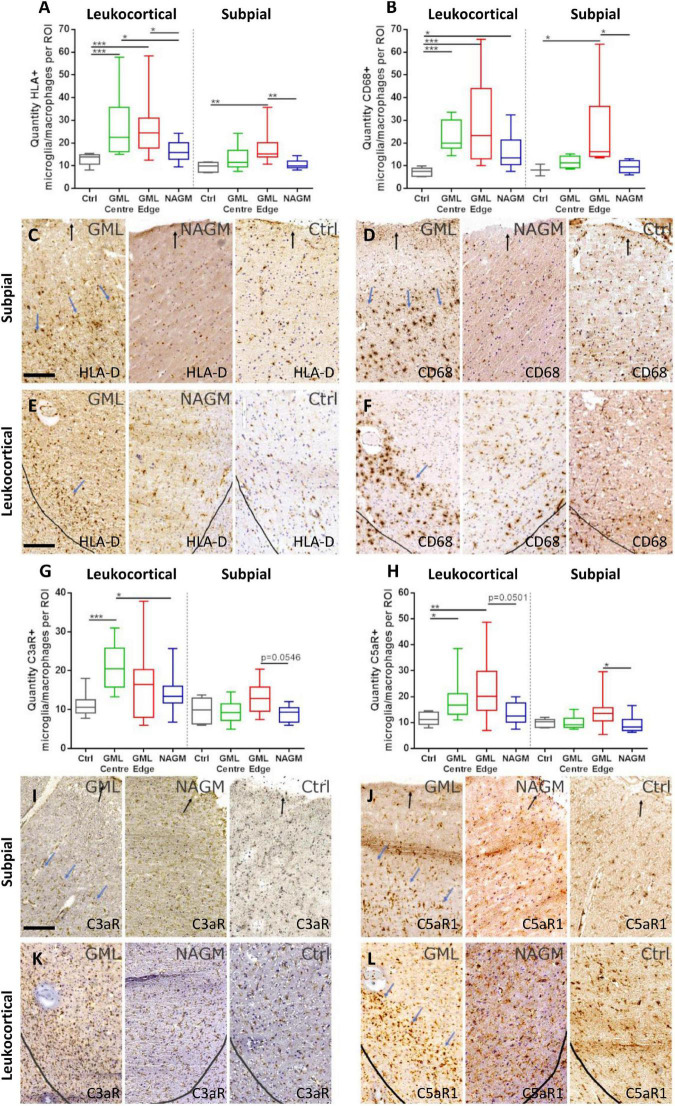
C5aR1 + microglia/macrophages are increased in density at the expanding grey matter lesion edge. Microglia/macrophages immunopositive for activation markers HLA-D and CD68 were quantified in leukocortical and subpial GM lesions **(A,B)**. The density of immunostained cells were significantly increased at the centre and edge, and at the edge of GM lesions, respectively, for both markers compared to non-neurological control GM at matched anatomical cortical level **(A,B)**. Examples of HLA-D **(C,E)** and CD68 **(D,F)** immunostaining in GM lesion (GML), normal appearing GM (NAGM), and control (ctrl). Quantification of C3aR1 + microglia/macrophages revealed a significant increase in quantity at the centre of deep cortical GM lesions compared to controls and NAGM and there were no significant changes in subpial GM lesions **(G,I,K)**. C5aR1 + microglia/macrophages were significantly elevated at the GM lesion centre and edge of leukocortical GM lesions compared to control GM, and at the GM lesion edge of subpial lesions compared to NAGM **(H,J,L)**. Blue arrows represent the expanding lesion edge of leukocortical and subpial lesions. Black arrows show the pial surface and grey lines represent the grey/white matter border. Data compared by Kruskal–Wallis and Dunn’s multiple comparison post-test. Scale bars = 100 μm.

We next sought to quantify the density of C3aR1 and C5aR1+ cells in subpial and leukocortical lesions (Figure 5). The density of HLA-D+ and CD68+ microglia/macrophages was increased at the lesion centre and edge of deep lying (leukocortical) GM lesions in comparison to anatomically matched control GM (Figures 5A, B), reflecting the chronic active inflammatory component of many of these lesions (Figures 5A–F). The density of HLA-D+ and CD68 microglia/macrophages was also increased at the edge of subpial lesions in comparison to control GM. The number of C3aR1+ microglia/macrophages per field of view were elevated at the GM lesion centre in comparison to control GM and normal appearing GM. There was no significant difference in the density of C3aR1+ microglia/macrophages between the different regions of interest in the subpial GM (Figures 5G, I, K).

The greatest increase in quantity of C5aR1+ microglia/macrophages was noted at the GM lesion edge of both leukocortical and subpial GM lesions, where additionally, the number of C5aR1+ cells also differed between the leukocortical GM lesion centre and control GM (Figures 5H, J, L).

### Complement anaphylatoxin receptor positive microglia/macrophages are associated with myelin and neuronal damage

**FIGURE 6:**
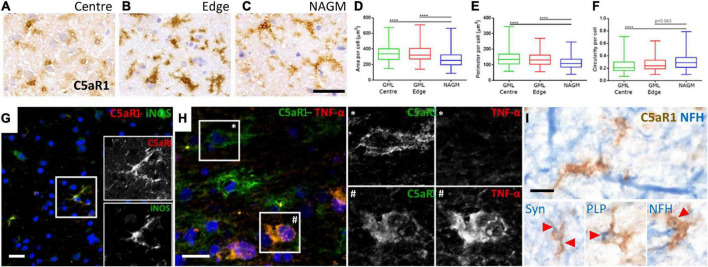
Activated C5aR1 + microglia/macrophages in cortical grey matter lesions. C5aR1 + microglia/macrophages in the centre or at the edge of subpial lesions display an altered morphology **(A–C)** and were quantifiably larger in area **(D)**, with a smaller perimeter **(E)**, and were more circular **(F)**, in comparison to C5aR1 + cells of the paired normal appearing GM (NAGM). C5aR1 + microglia/macrophages in a chronic active cortical GM lesion (case MS438) demonstrating dual-immunostaining for iNOS (**G** and inset), and TNFα (**H** and inset *, #). C5aR1 + microglia-like cells (brown immunostain) in close apposition to neurofilament-H + neurites (blue reaction product; **I**) or associated with inclusions of neuronal (synaptophysin, Syn +) and myelin (PLP +) products of degeneration. Scale bars, panels **(A–H)** = 25 μm; panel **(I)** = 10 μm.

Microglia/macrophages undergo morphological and phenotypic alterations in disease settings. We investigated the morphology of C5aR1+ microglia/macrophages at the cortical GM lesion edge and centre in comparison to the paired normal appearing GM from the same case (Figures 6A–C). C5aR1+ microglia/macrophages had an altered morphology; they were larger, with a greater perimeter and more rounded (more macrophage-like) in GM lesion edge and centre regions in comparison to anatomically matched normal appearing GM (Figures 6D–F). C5aR1+ microglia/macrophages were immunopositive for inducible nitic oxide synthase (iNOS; 6G and inset), TNFα (Figure 6H and inset), and those at or near inflammatory demyelinating lesions of the progressive MS cortex were associated with inclusions of debris-like material that stained immunopositive for PLP, non-phosphorylated neurofilament (heavy chain) and synaptophysin (Figure 6I).

## Discussion

We describe the presence of products of complement activation in the leptomeninges, perivascular spaces and parenchyma of MS characterised by extensive cortical demyelination and compartmentalised inflammation. The presence of complement activation proteins from both classical and alternative pathways implies complement activation at the CSF-brain barriers is ongoing throughout the disease process, which was in excess of 30 years in some cases. The localisation of complement activation proteins on damaged neurons, myelin and synapses is well described in the MS literature, but the relationship between complement activation at the brain barriers and changes in parenchymal, resident cell populations, such as microglia/macrophages, and the severity of GM and WM pathology is not clearly defined. Complement anaphylatoxin receptor positive cells (C3aR1 and C5aR1) were increased in density at, or near, cortical demyelinating lesions. C5aR1+ microglia/macrophages associated with ongoing expansion of both subpial and leukocortical GM lesions, displayed an altered morphology, expressed TNFα, iNOS and contained puncta immunoreactive for PLP, neurofilament and synaptophysin antigens. The generation of anaphylatoxin peptides associated with activated, proinflammatory microglia/macrophages and ongoing phagocytosis of neural and oligodendrocyte membranes suggests the C5a/C5aR1 axis may represent a treatment target to abrogate compartmentalised inflammation and cortical pathology in progressive MS.

Compartmentalised inflammation drives disease worsening independently of acute clinical relapses and inflammatory lesions (Cree et al., 2019). The compartmentalised inflammatory response is composed of activated immune cells in the parenchyma, reactive microglia and astroglia, increased numbers of leptomeningeal and connective tissue T and B lymphocytes, long-lived plasma cells, and elevated levels of circulating cytokines in the CSF (Lassmann, 2019). An overtly inflammatory CSF cytokine profile is associated with a more severe MS at presentation and follow-up, and pathologically, is linked to subpial tissue damage in a gradient-like pattern, which can be replicated in experimental models by the induced expression of inflammatory cytokines in the leptomeninges (Magliozzi et al., 2010, 2018, 2020; Gardner et al., 2013; James Bates et al., 2022). The levels of complement proteins, including C1q, C2, C4, and C5 in MS CSF and serum are altered in comparison to healthy individuals and between different MS clinical subtypes (Sellebjerg et al., 1998; Jongen et al., 2000; Finehout et al., 2005; Stoop et al., 2008; Ingram et al., 2010; Aeinehband et al., 2015; Magliozzi et al., 2019a; Zelek et al., 2019). Complement, together with serum proteins, including fibrin, represent effector proinflammatory molecules with the potential to signal across the CSF- brain barriers to activate microglia and astrocytes (Faustmann et al., 1995; Davalos et al., 2012; Ryu et al., 2015; Lee et al., 2018). Only a small number of prior studies have investigated the presence of complement proteins and activation products at the key CSF- and blood- brain barrier sites of pathological samples of MS (Wayne Moore et al., 2016; Guo et al., 2017). We show the widespread localisation of C1q, C4d, Bb, and C3b at the choroid plexus, ventricular ependyma and a relative increase at leptomeningeal and parenchymal vessels, which associated with the extent of cortical GM pathology. Therefore, complement deposition and activation at the pia and perivascular sites represents a component of the compartmentalised inflammatory response in MS, which is positioned to stimulate inflammation, lead to disruption of essential brain barriers, and support the formation or expansion of GM lesions by triggering local microglia and astrocytes.

The relative extent of complement immunoreactivity correlated modestly with the degree of leptomeningeal cellular infiltration and the extent of cortical GM demyelination (Figure 2), whilst parenchymal C3b immunoreactivity has been associated with activated microglia in the medial thalamus, another structure which lies close to CSF-filled spaces in the brain (Cooze et al., 2022). Infiltrating lymphocytes are a source of complement proteins in the subarachnoid space (Stahel et al., 1997) and the density of leptomeningeal infiltrates correlates closely with the degree of inflammation, demyelination, and neuro-axonal loss in the MS neocortex, cerebellum, and spinal cord (Magliozzi et al., 2007; Howell et al., 2011, 2015; Reali et al., 2020). The intrathecal activation of the classical (C1q, C4d) and alternative (Bb) pathways generates C3- and C5-convertases essential for the synthesis of the potent soluble anaphylatoxin fragments, which are also elevated in MS CSF (Ingram et al., 2010; Håkansson et al., 2020). The soluble complement anaphylatoxins can activate chemotactic, protective and neuron damaging functional pathways in microglia/macrophages and other cells by engaging with C3aR1 and C5aR1 (Woodruff et al., 2006; Ingersoll et al., 2010; Pavlovski et al., 2012). In addition, C3b-iC3b on cell surfaces is the ligand for complement receptor 3 (CR3, CD11b, MAC-1)-mediated phagocytosis and is important in mediating processes of synapse removal during development and in disease (Salter and Stevens, 2017). The phagocytosis of neural membranes and synapses labelled with C1q-C3 by activated microglia is seen in MS and its models, where reducing complement activation or increasing its clearance, protects neurites and synapses and preserves neurological function (Hammond et al., 2020; Werneburg et al., 2020; Gharagozloo et al., 2021; Ramaglia et al., 2021). Therefore, complement activation in the vicinity of resident microglial could be an important component mediating the extensive damage and loss of synapses, neurites and neurons of the cortical GM, which is a key component to the irreversible clinical worsening of progressive MS (Peterson et al., 2001; Magliozzi et al., 2010; Jürgens et al., 2016).

Several studies have documented the increased number of C3aR1 and C5aR1 cells in MS lesions (Gasque et al., 1998; Ischenko et al., 1998; Ingram et al., 2014), including cortical GM lesions (Watkins et al., 2016; Loveless et al., 2018). However, the identity of the cells most widely expressing the anaphylatoxin receptors, or their relative contribution to the extent of inflammation and demyelination, has not been clarified. Alongside a robust expression on myeloid lineage cells (Klos et al., 2013), earlier reports demonstrated anaphylatoxin receptor expression (both C3aR1 and C5aR1) on neurons, astrocytes and endothelia (Gasque et al., 1997, 1998; Ischenko et al., 1998; Davoust et al., 1999; Stahel et al., 2000; Farkas et al., 2003; Brennan et al., 2015). In MS, the expression appears restricted to myeloid cells, including microglia, and activated astrocytes (Müller-Ladner et al., 1996; Ischenko et al., 1998). We confirmed C3aR1 and C5aR1 expression by HLA-D+ and TMEM119 microglia/macrophages. Note that TMEM119 expression is downregulated in activated, non-homeostatic microglia, such as those found at the chronic active lesion edge (Zrzavy et al., 2017; Vankriekelsvenne et al., 2022). We did not anaphylatoxin receptor expression on cortical astrocytes, cells that are phenotypically and morphologically diverse and distinct from astrocytes of the WM (Oberheim et al., 2009; Schirmer et al., 2019). We confirmed a specific microglia/macrophage pattern of C3aR1 and C5aR1 expression in primary mouse dissociated cultures and induced human microglia-like cells. Induced human microglia-like cells expressed TMEM119 and TREM2 and are a useful source of human-derived cells for the analysis of microglial/macrophage biology (Sellgren et al., 2019). Microglia expressing TREM2 are associated with phagocytosis of apoptotic neurons and the subsequent loss of homeostatic function and the adoption of a neurodegenerative phenotype, which associated with neuronal loss (Krasemann et al., 2017). Reducing TREM2 expression on microglia attenuates inflammation and is protective against neurodegenerative pathology (Leyns et al., 2017). Therefore, our demonstration of the expression of TREM2, TMEM119 together with C3aR/C5aR1, is consistent with a population of reactive microglia that have lost some homeostatic functions and acquired detrimental phenotypic features.

It is important to note that complement can elicit a range of protective as well as damaging responses in the CNS. For example, C3a/C3aR1 and C5a/C5aR1 axis activation is reported to protect cultured neurons from excitotoxic death (van Beek et al., 2001; Mukherjee et al., 2008) and is associated with the induced release of neurotrophins, cell survival and enhanced neurogenesis, in some settings (Jauneau et al., 2006; Haynes et al., 2013; Westacott et al., 2021); whilst C3a and C5a overexpressing mice displayed similar rates of remyelination (Ingersoll et al., 2010), although presenting larger and more inflammatory demyelinating lesions. More work clearly needs to be done to fully understand the role of C3aR1 and C5aR1 activation at all stages of MS. A third receptor C5aR2 (C5L2 or GPR77), is less well characterised and regarded as a scavenger or default receptor competing with C5aR for C5a binding. The functional role for C5aR2 and its relationship to the intracellular signalling pathways regulating inflammation remains to be determined (Ward, 2009; Klos et al., 2013); expression of C5aR2 was not investigated in this study.

The borders of leukocortical and subpial GM lesions were demarcated by elevated numbers of HLA-D and CD68 + cells, which were mirrored by an increased number of C3aR1 and C5aR1+ microglia/macrophages. Previously, we have shown that the number of C1q and Bb immunopositive cells in MS leukocortical lesions correlated with the density of HLA-D+ and C5aR1+ microglia (Watkins et al., 2016). Our current findings implicate circulating as well as locally generated complement, to be playing a role in driving pathology in both subpial and deeper cortical laminae. C5aR1+ microglia within a chronic active GM lesion demonstrated subtle morphological alterations consistent with a transition of process bearing microglia to a more rounded and simpler morphological state (Figure 5), reminiscent of IBA-1+ microglia that associate with a more profound cortical pathology in progressive MS (van Olst et al., 2021). C5aR1+ microglia/macrophages in MS cortical GM co-stained for iNOS and TNFα and demonstrated cytoplasmic PLP, neurofilament + and synaptophysin + phagocytosed material and were in close proximity to C3b+ stressed/damaged cortical neurons (Figure 3; Watkins et al., 2016), suggesting that some of these cells contribute to worsening tissue damage.

C5aR1 activation, *via* the induced production of interferon γ and TNFα by macrophages and other monocytes (Pandey et al., 2017; Serfecz et al., 2021), may be one mechanism by which activated complement elicits damage. The induced expression of such cytokines can be blocked with the brain penetrant C5aR1 inhibitor PMX53 (Serfecz et al., 2021). Other small molecule antagonists of C5aR1 are effective in abrogating neuroinflammation, inflammasome activation and neurodegeneration *in vivo*, are brain penetrant and non-toxic. Eculizumab, a monoclonal antibody approved for use in paroxysmal nocturnal hemoglobinuria, prevents C5 convertase cleaving C5 into C5a. Trials of eculizumab in neuromyelitis optica have been encouraging (Pittock et al., 2019). Avdoralimab, a monoclonal antibody designed to block C5a binding to C5aR1, can prevent neutrophil migration into lungs and blocked inflammatory cytokine release in COVID-19 infection (Woodruff and Shukla, 2020). Therefore, these distinct pharmaceutical approaches to reduce C5aR1 activation may represent useful mechanisms to modulate resident microglia and other C5aR1 expressing cells in MS (Vergunst et al., 2007; Michailidou et al., 2018; Carpanini et al., 2019; Schartz and Tenner, 2020).

Cultured astrocytes and microglia can be induced to synthesise complement components, including C1q, C3, and factor B, when exposed to CSF from lymphotoxin-α treated rats. Lymphotoxin-α is elevated in concentration in the MS CSF and when over-expressed in the leptomeninges, recapitulates the pattern of subpial demyelination seen in disease (James Bates et al., 2022). Therefore, complement activation in the connective tissue spaces and parenchyma can stimulate the production of TNFα and other pleiotropic cytokines, which can be directly cytotoxic (and differentially expressed in cases characterised by leptomeningeal inflammation and more extensive cortical demyelination) (Magliozzi et al., 2019b; Picon et al., 2021), whilst cytokine release by activated glia can further drive the transcription of genes encoding early complement proteins. Future work, for example, could assess the effect of blocking C5aR1 activation in models of leptomeningeal inflammation and subpial cortical demyelination.

Microglia can induce a reactive astrocyte response *in vitro*, in part requiring IL-1β, TNFα and C1q, that is associated with a neuroinflammatory and non-homeostatic response and the elevated transcription of C3 and other signature genes (Liddelow et al., 2017). C3+ astrocytes are seen in actively demyelinating MS WM lesions and C3b+ astrocytes, some with a dysmorphic appearance, were frequently observed in subpial and peri-vascular sites in our MS GM (Supplementary Figure 2). The expression of C3 is not by itself an indicator of the neurotoxic potential of an astrocyte and such findings should be treated cautiously (Escartin et al., 2021). Indeed, we found the density of C3b+ astrocytes were unchanged between control and MS, whilst conversely, the density of C3b-/GFAP+ astrocytes were significantly reduced at subpial and perivascular GM lesions. These data demonstrate alterations in astrocytes occur in association with complement activation at or near the cortical GM.

Our current study is limited by the non-availability of neuroinflammatory disease control tissues harbouring frank inflammatory lesions in the selected blocks of interest, which were chosen as they anatomically matched the available MS blocks. A further limitation was that the assessment of complement immunostaining was performed semi-quantitatively and was not automated. The presence of staining artefacts of the delicate leptomeninges could be recognised by the investigator but was challenging to quantify digitally.

## Conclusion

The presence of elevated levels of complement C1q, C3b, C4d, and Bb, and the release of anaphylatoxins that engage C3aR1 and C5aR1 expressed by microglia, may stimulate functional pathways leading to the induced expression of inflammatory cytokines and phagocytic processes in the MS grey matter. Therefore, complement activation of anaphylatoxin receptor bearing microglia/macrophages represents a modifiable pathway to abrogate the extent of compartmentalised inflammation and cortical grey matter pathology to improve the outcome for some people with progressive MS.

## Data availability statement

The original contributions presented in this study are included in the article/Supplementary material, further inquiries can be directed to the corresponding author.

## Ethics statement

The studies involving human participants were reviewed and approved by the South Wales Research Ethics Committee (study numbers 08/MRE09/31+5 and 13/WA/0292). The patients/participants provided their written informed consent to participate in this study.

## Author contributions

RE, LW, BM, JN, and OH: methodology, investigation, writing – original draft, review and editing, and funding. KH, GS, CD, MN, and MD: methodology, investigation, and writing – review and editing. MR and DF: resources and writing – review and editing. All authors contributed to the article and approved the submitted version.

## Footnotes

1
https://qupath.github.io/

2
https://imagej.nih.gov/ij/index.html

3
https://imagej.net/downloads
