# Supplementary material for: Complement activation and increased anaphylatoxin receptor expression are associated with cortical grey matter lesions and the compartmentalised inflammatory response of multiple sclerosis
Source: Front Cell Neurosci. 2023 Mar 22;17:1094106. doi: 10.3389/fncel.2023.1094106 (PMC10073739; doi:10.3389/fncel.2023.1094106)
Supplement: Supplementary file 1 [file Data_Sheet_1.PDF]

Supplementary table 1:

	Relative extent of complement immunoreactivity				
	1	2	3	4	5
**Meninges/ subpia**	Lepto-meningeal/ pia	1 + meningeal vessels	2 + 5-20 cells	2 + 21-30 cells	2 + >30 cells
**P'vasc GM**	Vessel wall	1 + cell staining	2 + <5 cells	2 + 6-10 cells	2 + >10 cells
**P'vasc WM**	Vessel wall	1 + cell staining	2 + <15 cells	2 + 16-30 cells	2 + >30 cells

Supplementary table 1: Assessing complement immunoreactivity in the leptomeninges and brain parenchyma. Rating system used to assess relative extent of complement immunoreactivity at the leptomeninges, pia and underlying subpial tissues (row 1); blood vessels and surrounding parenchyma of the cortical GM (row 2); and blood vessels and surrounding tissue of the sub-cortical WM (row 3). A low rating of relative complement immunoreactivity (i.e. rated 0-1) corresponded to staining of surface or lining of the tissues only. Cases with a medium rating (rated 2-3) displayed connective tissue staining and small numbers of meningeal or perivascular cells stained for the complement protein of interest. Cases with a high complement rating (rated 4-5) displayed positive cell staining in the connective tissue spaces and extending to the subpial cortical laminae or surrounding parenchymal tissues, respectively. Abbreviations; GM, grey matter; WM, white matter.
