# Supplementary figures and images for: Complement activation and increased anaphylatoxin receptor expression are associated with cortical grey matter lesions and the compartmentalised inflammatory response of multiple sclerosis
Source: Front Cell Neurosci. 2023 Mar 22;17:1094106. doi: 10.3389/fncel.2023.1094106 (PMC10073739; doi:10.3389/fncel.2023.1094106)

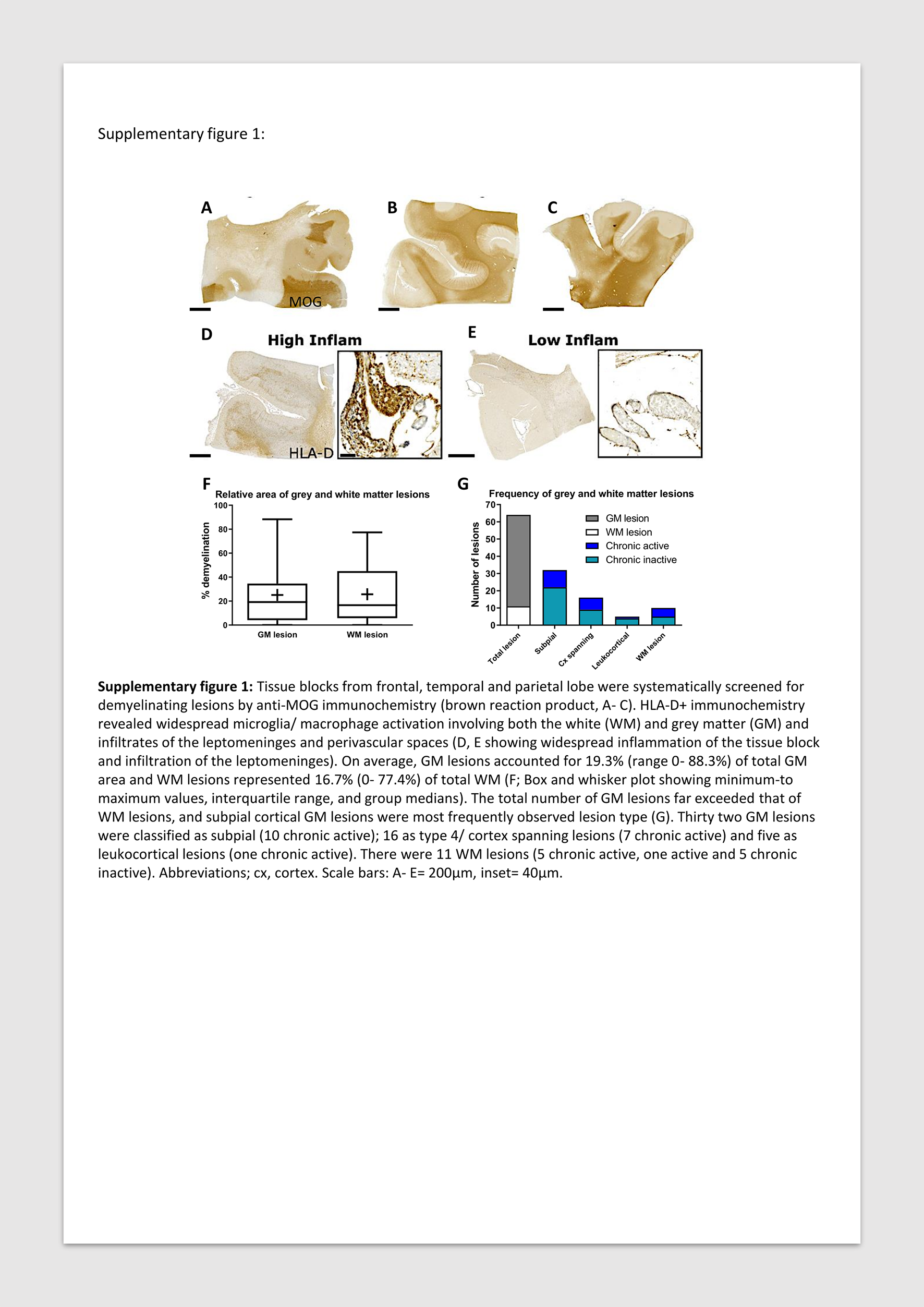

Supplement: Supplementary file 2 [file Image_1.TIFF]
