# Supplementary material for: Complement activation and increased anaphylatoxin receptor expression are associated with cortical grey matter lesions and the compartmentalised inflammatory response of multiple sclerosis
Source: Front Cell Neurosci. 2023 Mar 22;17:1094106. doi: 10.3389/fncel.2023.1094106 (PMC10073739; doi:10.3389/fncel.2023.1094106)
Supplement: Supplementary file 3 [file Image_2.pdf]

Supplementary figure 2:

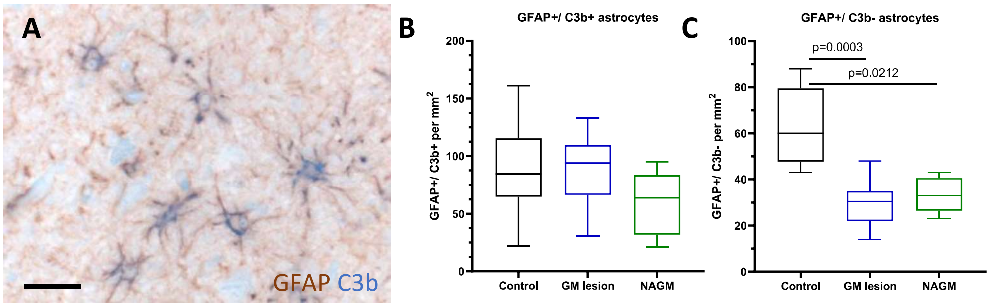

Supplementary figure 2: Density of GFAP/ C3b+ dual-stained astrocytes in control, MS cortical GM lesion and normal appearing GM (A). No difference in the density of GFAP/ C3b+ astrocytes was seen between control and MS GM lesions or normal appearing GM (NAGM; B). Quantification of the mean density of GFAP+/ C3b- astrocytes revealed a reduction in the number of GFAP+/ C3b- astrocytes in GM lesion and NAGM in comparison to controls. Kruskal-Wallis and Dunn's post-test. Box and whisker plot showing minimum-to maximum values, interquartile range, and group medians. Scale bar= 20µm.
