# Supplementary material for: Complement activation and increased anaphylatoxin receptor expression are associated with cortical grey matter lesions and the compartmentalised inflammatory response of multiple sclerosis
Source: Front Cell Neurosci. 2023 Mar 22;17:1094106. doi: 10.3389/fncel.2023.1094106 (PMC10073739; doi:10.3389/fncel.2023.1094106)
Supplement: Supplementary file 4 [file Image_3.pdf]

Supplementary figure 3:

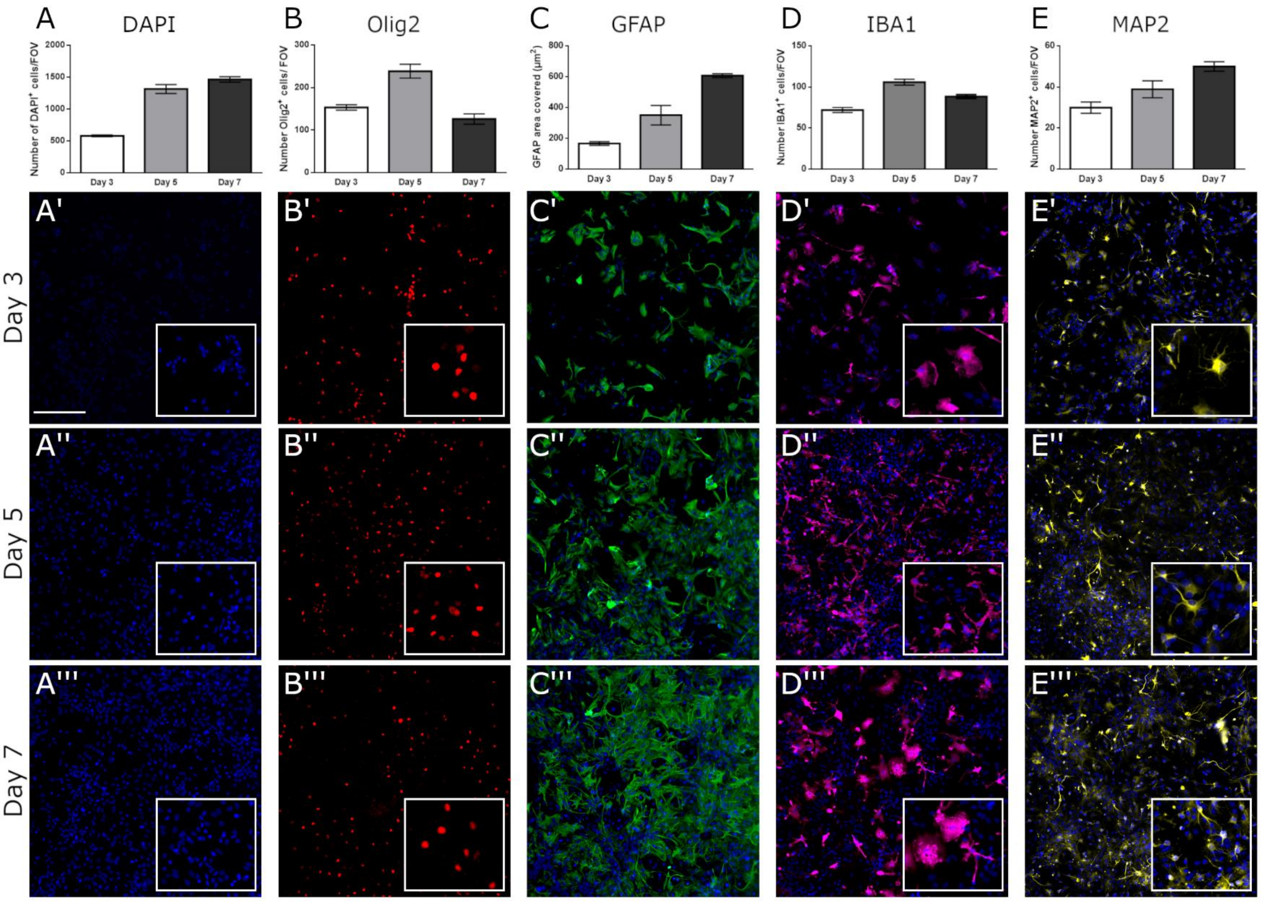

Supplementary figure 3: Characterisation of cell populations at different time points in primary murine dissociated cultures. Primary cultures were immune stained with anti- Olig2 (B’-B’’’), GFAP (C’-C’’’), IBA1 (D’-D’’’) and MAP2 (E’-E’’’) and each marker manually or automatically quantified. Total number of cells (DAPI+ nuclei) increased from culture day 3 to 5, with little difference in total number of cells between day 5 and 7 (A). GFAP area and MAP2+ cell quantity increased over time (C & E), whereas IBA1+ cells peaked in quantity at day 5 in culture with marginal decrease at day 7. Morphologically, IBA1+ microglia appeared macrophage- like at day 3, became smaller and more ramified at day 5, and were larger and more ameboid at day 7 (inserts, D’-D’’’). Scale bar 200µm (A’, applicable to all images).
